# Epstein–Barr Virus DNA Exacerbates Colitis Symptoms in a Mouse Model of Inflammatory Bowel Disease

**DOI:** 10.3390/v13071272

**Published:** 2021-06-29

**Authors:** Sirine Andari, Hadi Hussein, Sukayna Fadlallah, Abdo R. Jurjus, Margret Shirinian, Jana G. Hashash, Elias A. Rahal

**Affiliations:** 1 Department of Experimental Pathology, Immunology and Microbiology, American University of Beirut, Beirut, Lebanon; sia25@mail.aub.edu (S.A.); hmh70@mail.aub.edu (H.H.); smf28@mail.aub.edu (S.F.); ms241@aub.edu.lb (M.S.); 2 Center for Infectious Diseases Research, American University of Beirut, Beirut, Lebanon; 3 Department of Anatomy, Cell Biology and Physiological Sciences, Faculty of Medicine, American University of Beirut, Beirut, Lebanon; aj00@aub.edu.lb; 4 Division of Gastroenterology, American University of Beirut Medical Center, Beirut, Lebanon; ja38@aub.edu.lb

**Keywords:** inflammatory bowel disease, Epstein–Barr virus, IL-17A, Toll-like Receptors

## Abstract

Infection with EBV has been associated with various inflammatory disorders including inflammatory bowel diseases (IBD). Contribution of this virus to intestinal disease processes has not been assessed. We previously detected that EBV DNA triggers proinflammatory responses via the activation of endosomal Toll-like receptor (TLR) signaling. Hence, to examine the colitogenic potential of EBV DNA, we used the dextran sodium sulfate (DSS) mouse colitis model. C57BL/6J mice received either DSS-containing or regular drinking water. Mice were then administered EBV DNA by rectal gavage. Administration of EBV DNA to the DSS-fed mice aggravated colonic disease activity as well as increased the damage to the colon histologic architecture. Moreover, we observed enhanced expression of IL-17A, IFNγ and TNFα in colon tissues from the colitis mice (DSS-treated) given the EBV DNA compared to the other groups. This group also had a marked decrease in expression of the CTLA4 immunoregulatory marker. On the other hand, we observed enhanced expression of endosomal TLRs in colon tissues from the EBV DNA-treated colitis mice. These findings indicate that EBV DNA exacerbates proinflammatory responses in colitis. The ubiquity of EBV in the population indicates that possible similar responses may be of pertinence in a relevant proportion of IBD patients.

## 1. Introduction

Inflammatory Bowel Disease (IBD) is a chronic inflammatory syndrome characterized by recurrent episodes of gastrointestinal inflammation. It affects more than 10 million people around the world and its incidence is on the rise [1]. IBD has an unclear multifactorial pathophysiology thought to involve the convergence of genetic predisposing factors, immune system dysregulations, environmental triggers and intestinal microbiota alterations. The Epstein–Barr virus (EBV) has been detected in multiple inflammatory gastrointestinal conditions including the two major types of IBD, Crohn’s disease and ulcerative colitis. EBV is a member of the *Herpesviridae* family of viruses that establishes life-long latency in the host [2]. EBV infects the majority of the world population and is a virus that commonly undergoes reactivation after primary infection and establishment of latency [3,4,5,6]. Primary infection with EBV is usually asymptomatic in childhood but causes infectious mononucleosis in adulthood [7]. Infection with EBV is associated with many types of cancers and various autoimmune diseases including rheumatoid arthritis (RA), multiple sclerosis and systemic lupus erythematosus [8]. This virus was reported to be present in the colon tissues of up to 44% of healthy controls but in 60–76% of colon tissues of IBD subjects with consequent odds ratios ranging from about 6 [9,10,11] to as high as 42 [12]. Levels of EBV detected in IBD inflamed tissues exceed what is expected based on the number of B lymphocytes-cells that harbor the virus-present in these tissues; while both the EBV viral load and the number of B lymphocytes are elevated in these inflamed tissues, these elevations are not proportionate to one another. Moreover, the detection of EBV viral lytic proteins, BamHI-M rightward reading frame number 1 (BMRF1) and BamHI-Z leftward reading frame number 1 (BZLF1), expressed in some colon tissues from ulcerative patients raises the possibility that active viral replication plays a role in perpetuating the gastrointestinal inflammation [13]. Therefore, several aspects of the involvement of EBV in these diseases remain to be elucidated including mechanisms it triggers in the host resulting in possible disease exacerbation.

We previously detected that DNA from this virus triggers the production of proinflammatory mediators such as IL-17A in mice [14] via the activation of endosomal Toll-like receptor 3, 7 and 9 (TLR) signaling [15]. A bacterial DNA control was not capable of triggering such responses. Moreover, we reported a correlation between the EBV viral load in blood and levels of IL-17A in RA patients [16] indicating that these proinflammatory responses to the virus are relevant in human disease states. With EBV undergoing frequent reactivations in the host tissue, its DNA, which has particular Th17-promoting capabilities as demonstrated by our studies, is shed likely resulting in aggravation of inflammatory conditions. We recently utilized the *Drosophila melanogaster* fly model to evaluate whether DNA from this virus contributes to inflammation and gut tissue damage [17,18]. We observed that flies that were fed EBV DNA after induction of gut injury with dextran sodium sulfate (DSS) had a significantly higher extent of hindgut inflammation compared to those administered DSS alone. Whether such an exacerbatory role is exhibited by EBV DNA in a complex mammalian system, such as in mice, remains to be investigated. Therefore, the main objective of this study was to determine the effect of EBV DNA on the severity of intestinal inflammation in a murine model of acute colitis and to determine potential underlying mechanisms contributing to this effect.

## 2. Materials and Methods

### 2.1. Induction of Acute DSS Colitis in C57BL/6J Mice and Treatment Administration

Acute colitis was induced in C57BL/6J mice as previously established by Okayasu et al. [19]. Female, 6–8-week-old C57BL/6J mice were used in the study. Mice were obtained from the Animal Care Facility at the American University of Beirut (AUB). 

**Figure 1:**
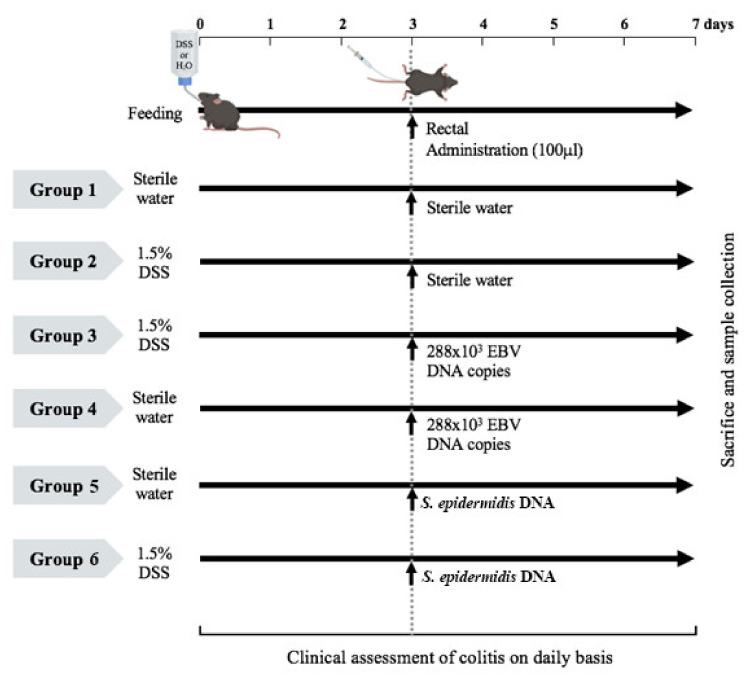
Experimental design used to assess the effect of Epstein–Barr virus (EBV) DNA on colitis severity in C57BL/6J mice. C57BL/6J mice (*n* = 9–14 per group) received either 1.5% dextran sodium sulfate (DSS)-containing or regular drinking water for 7 days. The three DSS-treated groups were then rectally administered sterile water, Epstein–Barr virus DNA in sterile water or *S. epidermidis* DNA in sterile water on day 3. The other normal drinking water-fed groups were included as controls and also received sterile water, EBV DNA or *S. epidermidis* DNA by rectal gavage on day 3.

To assess the effect of EBV DNA on the severity of colitis in mice, mice were allocated into six groups (Figure 1). Group one included nine mice and served as a negative control whereby mice received 100 µL of sterile water (the DNA diluent) by rectal gavage to control for any inflammatory responses that might result from the method of DNA administration and had no DSS added to their sterilized drinking water throughout the experimental period. Acute colitis was induced by oral administration of 1.5% (*w*/*v*) DSS (molecular weight 40 kDa; Chondrex, Redmond, WA, USA) in autoclaved drinking water ad libitum from day 0 to 7 in the second group of fourteen mice that were also rectally given 100 µL of sterile water on day 3. In group three, fourteen mice were treated with 1.5% DSS in the same manner as in group two but rectally received EDV DNA (Vircell, Granada, Spain) as a dose of 288 × 10^3^ DNA copies in 100 µL of sterile water on day 3 after initiation of DSS treatment. The fourth group included nine mice that received EBV DNA by rectal gavage, together with oral intake of drinking water from day 0 without being treated with DSS. The fifth group included nine mice that received 54.4 pg of *Staphylococcus epidermidis* DNA (equivalent to the weight of 288 × 10^3^ copies of EBV DNA) in 100 µL of sterile water by rectal gavage on day 3, together with oral intake of drinking water that did not harbor DSS. Group six on the other hand, included nine mice that were given drinking water containing 1.5% DSS and that received on 54.4 pg of *S. epidermidis* DNA in 100 µL of sterile water by rectal gavage on day 3 after DSS treatment initiation. The DSS dose selected was based on optimization experiments conducted to determine a level of colon injury that permitted possible further damaged induced by the other treatments. The EBV DNA dose was selected based on data from our studies examining systemic EBV and reported EBV levels in colonic tissues. *S. epidermidis* DNA was employed as a non-viral DNA control to ensure that observations were not generic ones induced by any DNA.

### 2.2. Mouse Monitoring and Assessment

Mice were monitored for 7 days and evaluated on a daily basis for weight loss, stool consistency and presence of blood in stools. These criteria were used to calculated the Disease Activity Index as per Cooper et al. [20]. On day 7, mice were sacrificed by cardiac puncture after sedation with sevoflurane and their colons were excised. The full length of each mouse colon, expected to become shorter with increased severity of colitis, was measured. 

Then, harvested colons were washed with ice-cold phosphate-buffered saline (PBS). Colon tissues were fixed in 10% formaldehyde overnight followed by embedding in paraffin. Five micrometer sections from the colon distal end were deparaffinized with xylene and rehydrated by passaging in a series of decreasing ethanol washes (100%, 95% and 70%) followed by staining with hematoxylin and eosin. The histology score was then determined as previously described [21,22]. Briefly, the score was a composite of scoring inflammation severity (0 = none; 1 = mild; 2 = moderate; 3 = severe), inflammation extent (0 = none; 1 = mucosa; 2 = mucosa and submucosa; 3 = transmural); and crypt damage (0 = none; 1 = one-third damaged; 2 = two-thirds damaged; 3 = crypt loss with present surface epithelium; 4 = crypt and surface epithelium loss). Added scores hence ranged from 0 to 10.

### 2.3. Expression Studies

RNA was extracted [23,24,25,26] from colon tissues using Qiazol (Qiagen, Hilden, Germany) as per manufacturer recommendations. For this purpose, 1 mL of the Qiazol reagent was added to 100 mg of mouse colon tissue and the sample was then homogenized using a Biospec Tissue Tearor (Bartlesville, OK). Then, 200 µL of chloroform were added, incubated for 5 min followed by centrifugation at 12,000× *g* for 15 min. Then, 500 µL of isopropanol were added to the aqueous phase, incubated for 10 min followed by centrifugation at 12,000× *g* for 10 min. The pellet was then washed with 75% ethanol [27], air-dried and then dissolved in RNase-free water. cDNA was then synthesized using the QuantiTect reverse transcription kit (Qiagen, Hilden, Germany) following the manufacturer’s instructions. One µg of RNA from each sample were incubated at 42 °C for 2 min with the genomic DNA wipeout buffer provided with the kit; then, 6 µL of the reverse transcription master mix were added and the mixture was incubated for 15 min at 42 °C for reverse transcription. The reverse transcriptase in the master mix was then inactivated by incubation for 3 min at 95 °C. Real-time PCR was subsequently employed to detect the relative gene expression levels of IL-17A, IFNγ and TNFα as well as TLR3, 7 and 9 normalized to the expression of β-actin in each sample. β-actin was selected since it is a housekeeping gene. Real time detection was carried out using SYBR Green (Bio-rad, Hercules, CA, USA) and employing the Bio-Rad CFX96 Real Time System. For this purpose, 100 ng of cDNA were added to 5 µL SYBR Green Supermix, 150 pmoles of each primer and RNase-free water for a total reaction volume of 10 µL. Previously published primer pairs for IL-17A [16], IFNγ [28], TNFα [29], CTLA4 [16], TLR3 [30], TLR7 [31], TLR9 and β-actin [16] were obtained from Macrogen (Seoul, South Korea). The real time program consisted of 5 min at 95 °C followed by 40 cycles of 95 °C for 15 s and the annealing temperature for each primer pair for 30 s. Sequences and annealing temperatures for each primer pair are indicated in Table 1. Generated amplicon species and primer specificity were ensured using high resolution melting curve analysis with 0.5 °C temperature increments over a temperature range of 65–95 °C. Relative expression levels per sample were then determined by normalization to levels in the control group (given DSS-free drinking water and a rectal gavage of water). Levels were calculated using the ΔΔCt method.

### 2.4. Statistical Analysis

Statistical analysis was performed using the GraphPad Prism software. Significance of DAI and histology score differences were evaluated using the Mann–Whitney U test. The unpaired t-test was performed to assess the statistical significance of body weight changes, colon length measurements and expression levels. *p*-values less than 0.05 were considered statistically significant. 

## 3. Results

### 3.1. Increased Body Weight Loss in the EBV DNA-Treated Mouse Colitis Model

To examine the effect of EBV DNA in an IBD model, DSS-induced colitis in C57BL/6J mice was used. This is a widely utilized model with well-established protocols for monitoring and assessment. Mice were given 1.5% DSS in drinking water or DSS-free water from day 0 to day 7. On day 3 mice were given EBV DNA, water or *S. epidermidis* DNA (bacterial DNA control) by intracolonic rectal gavage (Figure 1). 

**Figure 2:**
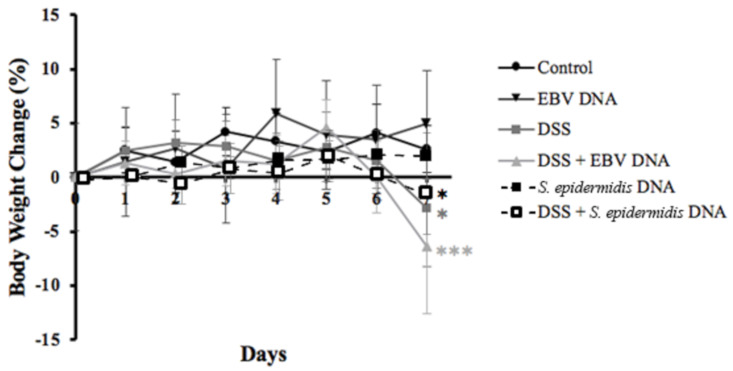
Average percent body weight change in control and experimental mouse groups used to assess the effect of Epstein–Barr virus (EBV) DNA on colitis severity in C57BL/6J mice. C57BL/6J mice (*n* = 9–14 per group) received either 1.5% dextran sodium sulfate (DSS)-containing or regular drinking water for 7 days. Three DSS-treated groups were then rectally administered sterile water, Epstein–Barr virus DNA in sterile water or *S. epidermidis* DNA in sterile water on day 3. The other normal drinking water-fed groups were included as controls and also received sterile water, EBV DNA or *S. epidermidis* DNA by rectal gavage on day 3. Mouse body weight was evaluated daily, and the percent body weight change was calculated per mouse per group compared to its initial weight on day 0. Average weight per group per time point and standard deviations are indicated. * *p* < 0.05, *** *p* < 0.001, compared to the control group on the respective day of measurement.

Initially, all mouse groups showed an average weight gain until day 5. This pattern of weight gain continued till the end of the monitoring period on day 7 in the normal and DNA control groups. In contrast, DSS treatment resulted in decreased average mouse body with the highest average body weight loss of 6.4% observed in the group that was fed on DSS and that received the EBV DNA treatment (*p* = 0.0005) (Figure 2). 

### 3.2. Enhanced Colon Shortening in the EBV DNA-Treated Mouse Colitis Model

**Figure 3:**
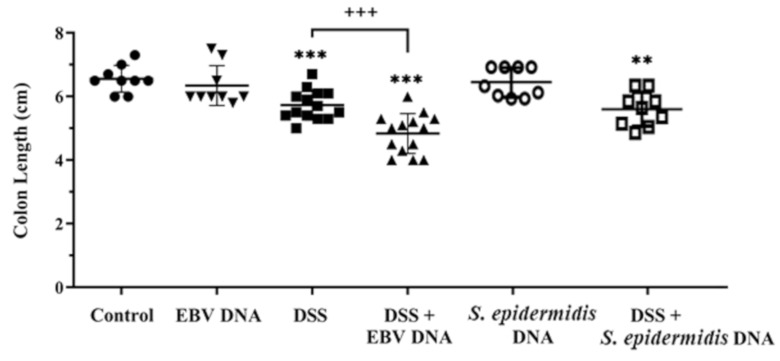
Average colon lengths in control and experimental mouse groups used to assess the effect of Epstein–Barr virus (EBV) DNA on colitis severity in C57BL/6J mice. C57BL/6J mice (*n* = 9–14 per group) received either 1.5% dextran sodium sulfate (DSS)-containing or regular drinking water for 7 days. Three DSS-treated groups were then rectally administered sterile water, Epstein–Barr virus DNA in sterile water or *S. epidermidis* DNA in sterile water on day 3. The other normal drinking water-fed groups were included as controls and also received sterile water, EBV DNA or *S. epidermidis* DNA by rectal gavage on day 3. On day 7 of the experimental protocol, mice were sacrificed and their colon lengths were measured. Average colon lengths per group and standard deviations are indicated. *** *p* < 0.001, compared to the control group; ** *p* < 0.01 compared to the control group; +++ *p* < 0.001, compared to the DSS group.

The severity of colitis in the IBD mouse model was also evaluated based on macroscopic examination of colon samples collected from each mouse group on day 7, a day frequently assessed in the acute colitis model, and examination for colon length shortening, which is a marker of inflammation. Colon lengths were significantly shorter in the three groups that received DSS compared to the normal water-fed control group; however, the highest extent of colon shortening was observed in the group that received EBV DNA in addition to DSS. The average colon length in the group that received DSS but no DNA treatments was 5.7 cm whereas it was 6.6 cm in the control group (*p* = 0.0003). On the other hand, the group administered DSS and EBV DNA had an average colon length of 4.8 cm (*p* = 0.0001) (Figure 3). Hence, the decrease in colon length in the group given DSS and EBV DNA was significant not only compared to the control group solely given water but also compared to the group that received DSS but no DNA treatments (*p* = 0.0002). The majority of mice in the group that received EBV DNA in addition to DSS had a colon length of 5.3 cm or shorter, while most of the mice that received DSS alone had a colon length of 5.3 cm or longer. The average colon length in the group given *S. epidermidis* DNA along with DSS in the drinking water was 5.6 cm and hence was on par with that of the group given DSS but no DNA treatments.

### 3.3. Exacerbated Disease Activity in the EBV DNA-Treated Mouse Colitis Model

**Figure 4:**
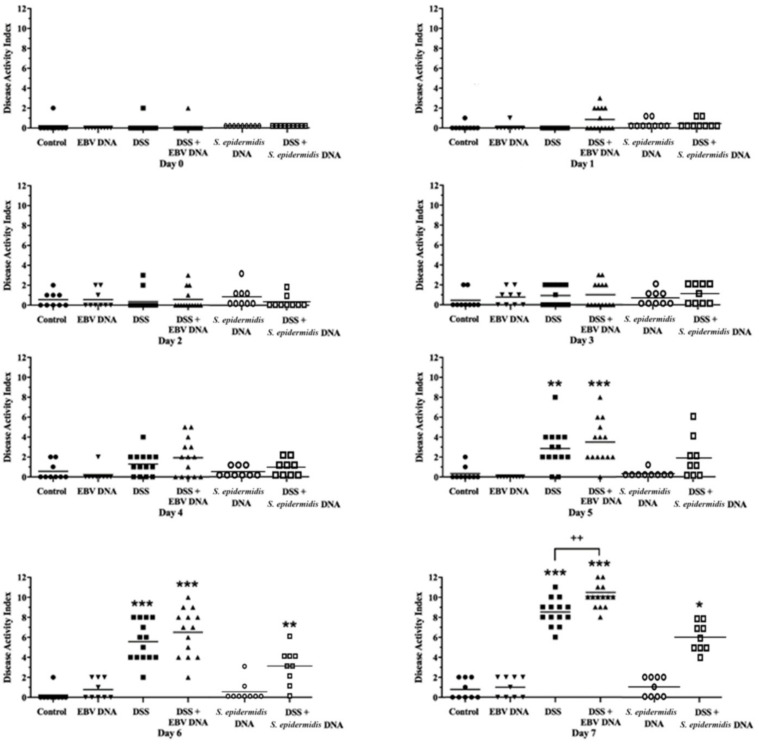
Disease activity index (DAI) scores in control and experimental mouse groups used to assess the effect of Epstein–Barr virus (EBV) DNA on colitis severity in C57BL/6J mice. C57BL/6J mice (*n* = 9–14 per group) received either 1.5% dextran sodium sulfate (DSS)-containing or regular drinking water for 7 days. Three DSS-treated groups were then rectally administered sterile water, Epstein–Barr virus DNA in sterile water or *S. epidermidis* DNA in sterile water on day 3. The other normal drinking water-fed groups were included as controls and also received sterile water, EBV DNA or *S. epidermidis* DNA by rectal gavage on day 3. The DAI was determined daily as a composite measure of the scores of body weight loss, stool consistency and fecal blood. Individual mouse and average DAIs per group are indicated. *** *p* < 0.05, ** *p* < 0.01, *** *p* < 0.001, compared to the control group on the same day; ++ *p* < 0.01, compared to the DSS group on the same day.

The Disease Activity Index (DAI) was calculated for the various groups as previously described [19]. The DAI score was increased in all DSS-administered groups compared to the normal water-fed group (Figure 4). Initially, the DAI scores gradually increased in these groups after day 2. By the end of the monitoring period, the scores observed in the group administered EBV DNA in addition to DSS were significantly higher (*p* = 0.0044) than those of the mice in the group that was fed on DSS but did not receive any DNA treatment. The DAI scores of all but one mouse in the group that received EBV DNA in addition to DSS was 9 or higher, whereas only half of the mice that received DSS alone had a DAI score of 9 or higher. While the scores in the group that received bacterial DNA in addition to DSS were higher than the control group, they were not higher than the group that only received DSS. Groups that were given DNA but no DSS treatments did not display any relevant disease activities. On the other hand, administration of DNA from human adenovirus type 1 subgroup C to mice fed on DSS resulted in a DAI similar to that of the mice fed on DSS in the absence of any DNA treatment (data not shown). Therefore, neither bacterial DNA nor viral non-EBV DNA were able to exacerbate the disease similar to EBV DNA. 

### 3.4. EBV DNA Administration Aggravates Histological Damage in the Colitis Model

**Figure 5:**
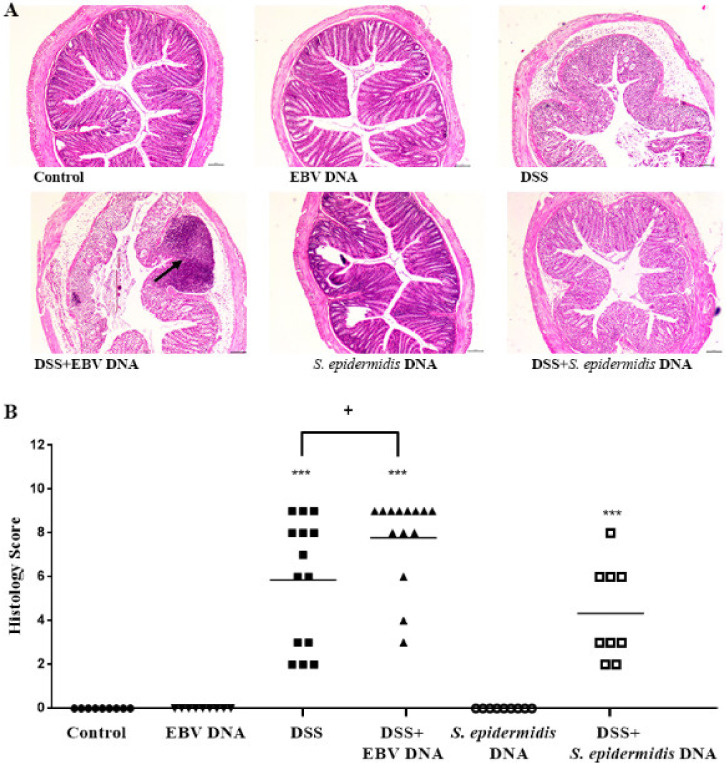
Histological damage in control and experimental mouse groups used to assess the effect of Epstein–Barr virus (EBV) DNA on colitis severity in C57BL/6J mice. C57BL/6J mice (*n* = 9–14 per group) received either 1.5% dextran sodium sulfate (DSS)-containing or regular drinking water for 7 days. Three DSS-treated groups were then rectally administered sterile water, Epstein–Barr virus DNA in sterile water or *S. epidermidis* DNA in sterile water on day 3. The other normal drinking water-fed groups were included as controls and also received sterile water, EBV DNA or *S. epidermidis* DNA by rectal gavage on day 3. On day 7 of the experimental protocol, mice were sacrificed, and their colons were collected. (**A**) Hematoxylin and eosin-stained distal sections of mouse colons. Arrow indicates an abscess. (**B**) Mouse group histology scores. Individual mouse scores and average group scores are indicated. *** *p* < 0.001, compared to the control group; + *p* < 0.05, compared to the DSS group.

Examination of colon tissue sections from the assessed mouse groups indicated increased crypt architecture damage, muscle thickening, and presence of abscesses in the DSS-fed groups (Figure 5A). The highest histological damage scores were observed in the group administered EBV DNA in addition to DSS (Figure 5B); the scores in this group were significantly higher than those of mice that were only given DSS (*p* = 0.0384). On the other hand, administration of bacterial DNA did not further the damage induced by DSS; the bacterial DNA rather lowered the histological damage score compared that of the DSS-fed group (*p* = 0.0203).

### 3.5. EBV DNA Enhances the Expression of Proinflammatory Mediators in the Colitis Model

**Figure 6:**
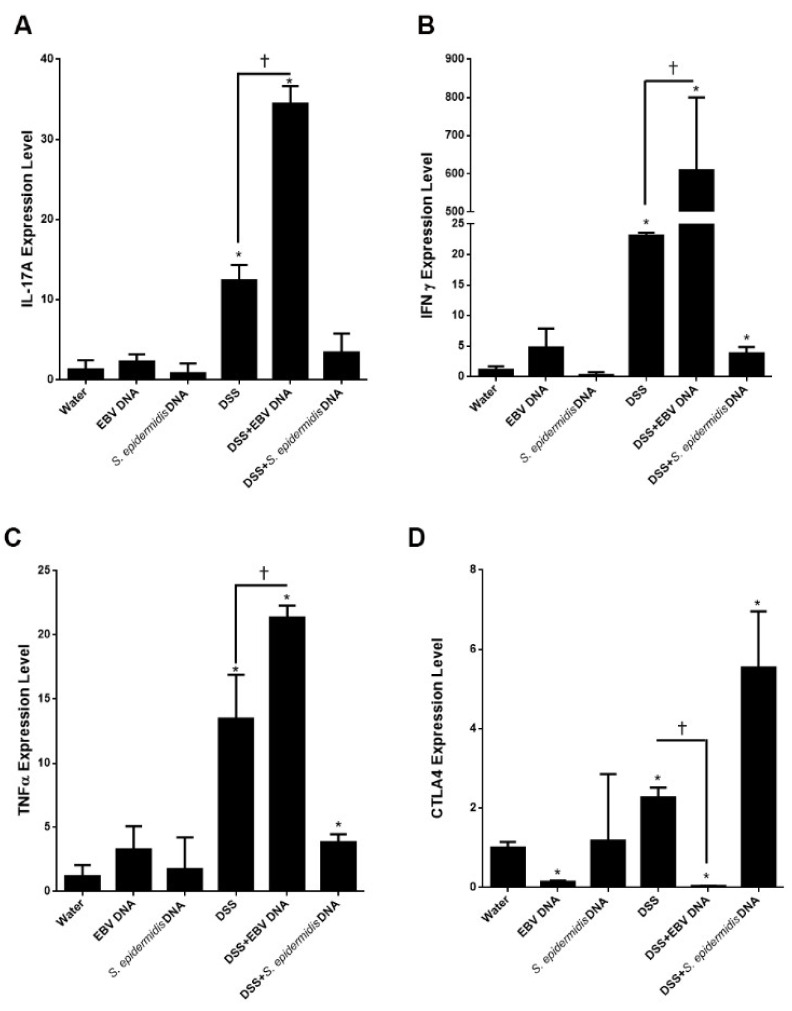
Expression levels of (**A**) IL-17A, (**B**) IFNγ, (**C**) TNFα and (**D**) CTLA4 in control and experimental mouse groups used to assess the effect of Epstein–Barr virus (EBV) DNA on colitis severity in C57BL/6J mice. C57BL/6J mice (*n* = 9–14 per group) received either 1.5% dextran sodium sulfate (DSS)-containing or regular drinking water for 7 days. Three DSS-treated groups were then rectally administered sterile water, Epstein–Barr virus DNA in sterile water or *S. epidermidis* DNA in sterile water on day 3. The other normal drinking water-fed groups were included as controls and also received sterile water, EBV DNA or *S. epidermidis* DNA by rectal gavage on day 3. On day 7 of the experimental protocol, mice were sacrificed, colons were collected and expression levels were determined in colon sections. Average expression levels per group and standard deviations are indicated. * *p* < 0.05, compared to the water-treated group; ^†^ *p* < 0.05, compared to the DSS-group.

We had previously described enhanced IL-17A levels in mice systemically treated with EBV DNA. To assess whether this mediator may underlie the aggravated disease activity seen in the colitis mice, we assessed its expression in mouse colon tissues (Figure 6A). IL-17A expression levels were markedly elevated in the DSS-fed group (*p* = 0.0009) and the EBV DNA-administered DSS-fed group (*p* = 0.0001) compared to the control. No significant changes were seen in the other groups. Notably, the level of IL-17A expression in the EBV DNA-administered DSS-fed group was 2.87-fold that of the group solely treated with DSS (*p* = 0.001). On the other hand, levels of both IFNγ (Figure 6B) and TNFα (Figure 6C) were notably higher in all groups given DSS in their drinking water with the highest increase being observed in the EBV DNA-administered DSS-fed group. Moreover, levels of both of these mediators were markedly higher in mice fed on DSS and given EBV compared to those solely given DSS. This correlates with the proinflammatory profile induced by EBV DNA that we had previously observed at the systemic level.

We also examined CTLA4 as an immunoregulatory marker (Figure 6D). CTLA4 has T cell suppressive roles and is expressed on relevant regulatory T cell populations as well as other types of T cells upon their activation [32]. Compared to the mock-treated control mice given only water, expression of this marker was significantly lower in the EBV DNA-treated group (*p* = 0.0005) and the EBV DNA-treated mice that were fed on DSS (*p* = 0.0006). In contrast, its levels were increased in the mice solely given DSS (*p* = 0.0062) or *S. epidermidis* DNA in addition to DSS (*p* = 0.0052). The highest elevation in the expression of CTLA4 was observed in the group given DSS along with the bacterial DNA, which may explain why this group had the lowest levels of proinflammatory marker expression among the DSS-administered mouse groups.

### 3.6. Enhanced Expression of Endosomal TLRs in EBV DNA-Treated Colitis Mice

**Figure 7:**
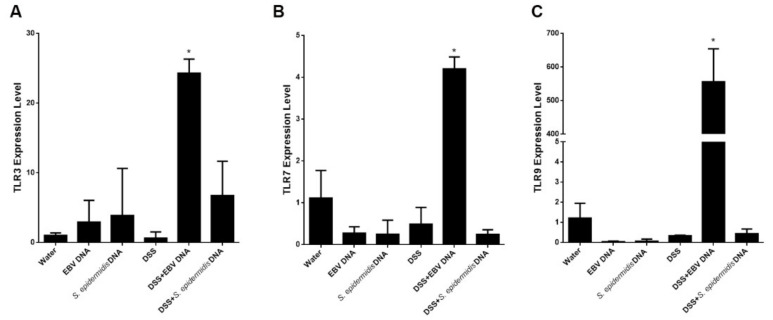
Expression levels of (**A**) TLR3, (**B**) TLR7 and (**C**) TLR9 in control and experimental mouse groups used to assess the effect of Epstein–Barr virus (EBV) DNA on colitis severity in C57BL/6J mice. C57BL/6J mice (*n* = 9–14 per group) received either 1.5% dextran sodium sulfate (DSS)-containing or regular drinking water for 7 days. Three DSS-treated groups were then rectally administered sterile water, Epstein–Barr virus DNA in sterile water or *S. epidermidis* DNA in sterile water on day 3. The other normal drinking water-fed groups were included as controls and also received sterile water, EBV DNA or *S. epidermidis* DNA by rectal gavage on day 3. On day 7 of the experimental protocol, mice were sacrificed, colons were collected and expression levels were determined in colon sections. Average expression levels per group and standard deviations are indicated. * *p* < 0.05, compared to the water-treated group.

We had previously demonstrated that the endosomal TLRs, TLR3, 7 and 9 played an essential role in mediating the increase in IL-17A induced by EBV DNA at the systemic level [15]. To examine whether the expression of these markers would also be affected at the tissue level upon treatment with EBV DNA, we examined their expression in mouse colon tissues (Figure 7). Unlike what we observed upon injection of mice with EBV DNA, expression levels of these TLRs did not increase upon treatment with EBV DNA alone. However, after feeding mice on DSS, EBV DNA resulted in significantly enhanced expression of TLR3 (*p* = 0.0002), TLR7 (*p* = 0.0091) and TLR9 (*p* = 0.0017) in colon tissues. This indicates that some level of injury is likely required for EBV DNA to induce these effects. The other assessed treatments did not result in relevant changes in the expression of these TLRs.

## 4. Discussion

Epidemiological studies show that more than 90% of the world population is seropositive for EBV [33,34]. Following the initial infection, the virus establishes latency in resting memory B cells with potential recurrent infections whereby viral DNA can be shed upon reactivation. Growing evidence confirms that reactivation of EBV after latency can occur at any mucosal site where B cells reside. Aside from the long-known associations of EBV with systemic lupus erythematosus, RA and multiple sclerosis, studies continue to demonstrate a possible implication of EBV in the pathogenesis of IBD [9,10,11,12]. However, the definitive role of EBV in IBD remains unclear and warrants further investigation. In the study at hand, we intended to examine the role played by DNA from this virus in colon inflammation; hence, to particularly examine the effects of the viral DNA in exacerbating inflammatory responses independent of other viral proteins or responses triggered by viral replication, we used a mouse colitis model. The mice employed are not permissive to EBV replication hence eliminating responses not pertaining to the DNA itself. Our studies indicate that EBV DNA plays a role in the exacerbation of colitis pathology by increasing the severity of the disease in a mouse model. The disruption of the epithelial barrier and the uncontrolled immune dysregulation that characterize DSS colitis induction in mice mirrors possible predisposing conditions that result in underlying damage in humans. This damage may provide a favorable environment for EBV DNA to instigate or aggravate IBD. Following primary infection in immunocompetent individuals, EBV persists for life in resting memory cells and escapes cytotoxic T cell surveillance by limiting viral gene expression. Possibly, the absence of immune surveillance that is associated with impaired mucosal immunity in IBD cases predisposes patients to reactivation of EBV infection resulting in increased mucosal inflammation and further disease perpetuation. Alternatively, minor mucosal damage in subjects with active EBV replication in their gastrointestinal tract may result in IBD due to the enhanced inflammation induced by the viral DNA. 

Subjects with Crohn’s disease were shown to have decreased macrophage activities and reduced neutrophil recruitment, which may allow passage of luminal content through the mucosa and impair bacterial clearance [35]. Dendritic cells, on the other hand, display enhanced activation in IBD with upregulated expression of microbial antigen receptors, such as TLR2 and TLR4, and activation markers, such as CD40 [36]; this results in activation of lymphocytic cells as well as endothelial cells, which leads to enhanced expression of inflammatory signals, cytokines and chemokines that mobilize the leukocytic compartment to the site of inflammation. Our previous studies indicating that EBV DNA favors a Th17-inclined proinflammatory profile at the systemic level were recapitulated here in colon tissues. We also observed enhanced expression of the nucleic acid-responsive endosomal TLRs. An enhanced T cell immune response has been reported in both Crohn’s disease and in ulcerative colitis. Whereas Crohn’s disease is believed to be more Th1-mediated and characterized by increased production of IFN-γ, a Th2-bias has been reported in ulcerative colitis despite evidence indicating that T cells from ulcerative colitis patients do not produce IL-4, the signature cytokine of the Th2 response [37]; however, increased production of IL-5, another Th2 cytokine, has been reported [38]. On the other hand, enhanced Th17 responses have been more associated with Crohn’s disease; nevertheless, detection of increased production of IL-17A, the hallmark cytokine of the Th17 response, has been observed in both intestinal tissue samples and sera from subjects with active Crohn’s disease and ulcerative colitis [39,40]. An anti-IL-17A monoclonal antibody tested as treatment for Crohn’s disease appeared to nevertheless enhance inflammation in a considerable number of patients [41]. This is possibly due to interference with a subset of Th17 cells that have a homeostatic role in the intestines rather than an inflammatory function; these cells play a role in enhancing the intestinal barrier capacity by stimulation of mucin production and regulation of barrier permeability among other functions [42]. These homeostatic Th17 cells are limited in their IFN-γ production and do not respond to pathogenic bacteria unlike inflammatory Th17 cells present in the intestines [42]. 

The particular aspects of the EBV genome resulting in enhanced inflammation in our model remain to be elucidated. The percent of genomic CpG dinucleotides in the EBV genome is about 10% whereas it is 4% in the *S. epidermidis* genome and 13% in the adenoviral genome. Hence, the variation seen in the response to these genomes in our studies may not be necessarily explained by the absolute percent of CpG motifs in these viral genomes but possibly by their methylation status as well as context within the genome itself. On the other hand, we observed a statistically significant decrease in IL-17A, IFNγ and TNF-α expression levels but an increase in CTLA4 levels in the *S. epidermidis* DNA + DSS group compared to the DSS-fed group. This may explain the decrease in histological damage as well as in the DAI seen in this group. The exact mechanism of amelioration remains to be elucidated.

Our data indicates that EBV DNA enhances colitis symptoms solely after DSS administration which signifies that this virus component does not instigate colitis by itself. Hence, these findings likely indicate that enhanced EBV replication and DNA generation does not induce IBD but rather worsens inflammatory responses possibly resulting in IBD flare-up episodes; this, nevertheless, remains to be tested in patients. Since, as indicated above, direct inhibition of IL-17A, the seemingly central mediator in the responses to EBV DNA as detected by our studies, enhances inflammation, using endosomal TLR pathway inhibitors may be an alternative route. We have observed enhanced expression of these mediators in our colitis model and have previously demonstrated [15] that inhibition of these TLRs decreases IL-17A production in response to EBV DNA. Hence, pending further investigations in human subjects, targeting endosomal TLR signaling may result in amelioration of disease symptoms without inhibition of IL-17A itself. Such treatments may be useful in subjects who test positive for viral replication markers, such as BZLF-1, which would indicate active production of the viral DNA. Hence, assessment of such replication markers may be of relevance in subjects with frequent flare-ups. 

Our studies hence indicate that EBV DNA increases the severity of colitis in a mouse model of IBD. Given the large swath of the population affected by this virus, our findings may have implications on possible preventative strategies, therapeutic approaches and measures taken to curtail flare-ups in IBD. While directly targeting IL-17A itself may have deleterious consequences in these conditions, targeting viral replication and DNA shedding, TLRs that seem to respond to the viral DNA or other mediators that play a role in this response may be of pertinence.

## Figures and Tables

**Table 1:** Primers and annealing temperatures used for real time PCR.

Gene	Primers	Annealing Temperature
TLR9	F: 5 ′-ACTGAGCACCCCTGCTTCTA-3′ R: 5 ′-AGATTAGTCAGCGGCAGGAA-3′	60 °C
TLR3	F: 5′-GGTGTTTCCAGACAATTGGCAAG-3′ R: 5′-TGGAGGTTGTTGTAGGAA-AGATCG-3′	60 °C
TLR7	F: 5′-CCACAGGCTCACCCATACTTC-3′ R: 5′-GGGATGTCCTAGGTGGTGACA-3′	60 °C
IL-17A	F: 5′-TTAAGGTTCTCTCCTCTGAA-3′ R: 5′-TAGGGAGCTAAATTATCCAA-3′	56 °C
CTLA4	F: 5′-GCCAGTGGTTCCAAAGGTTG-3′ R: 5′-CACTGTGGGACGACACTGAT-3′	60.9 °C
TNFα	F: 5′-AAATGGGCTCCCTCTCATCAGTTC-3′ R: 5′-CTGCTTGGTGGTTTGCTACGAC-3′	61 °C
IFNγ	F: 5′-TGAACGCTACACACTGCATCTTGG-3′ R: 5′-CGACTCCTTTTCCGCTTCCTGAG-3′	60 °C
β-actin	F: 5′-GGCATTGTTACCAACTGGGACGAC-3′ R: 5′-CCAGAGGCATACAGGGACAGCACAG-3′	58.6 °C

## Data Availability

All relevant data has been included within the manuscript.
